# Supplementary material for: Job satisfaction among healthcare workers in Ghana and Kenya during the COVID-19 pandemic: Role of perceived preparedness, stress, and burnout
Source: PLOS Glob Public Health. 2021 Oct 13;1(10):e0000022. doi: 10.1371/journal.pgph.0000022 (PMC10021773; doi:10.1371/journal.pgph.0000022)
Supplement: S1 Questionnaire — Sections of study questionnaire relevant to this manuscript. (PDF) [file pgph.0000022.s001.pdf]

S1 Questionnaire: Sections of study questionnaire relevant to this manuscript

Start of Block: Demographics

position 1. What is your current position?

- ◯ 0, Doctor (House officer/Medical officer/SMO) (1)
- ◯ 1, Doctor (Resident/Specialist/Consultant) (2)
- ◯ 2, Medical/Physician Assistant (3)
- ◯ 3, Nurse (4)
- ◯ 4, Midwife (5)
- ◯ 5, Auxilary clinical staff (e.g., Nurse aid) (6)
- ◯ 6, Medical Laboratory professional (7)
- ◯ 7, Pharmacist (8)
- ◯ 8, Administrator (9)
- ◯ 9, Other (specify) (10) ____

hf_type 2. What type of facility do you mostly work in?

- ◯ 0, Teaching Hospital (1)
- ◯ 1, Regional Hospital (2)
- ◯ 2, District Hospital / Polyclinic (3)
- ◯ 3, Gov't. Health Center (4)
- ◯ 4, Other Gov't Facility (5)
- ◯ 5, Mission Hospital (6)
- ◯ 6, Private Hospital / Clinic (7)
- ◯ 7, Other_Specify (8) ____

length_proovider
4. How many years have you worked as a health provider? (ENTER A NUMBER FROM 0 TO 99)

____

gender 5. How do you identify?

- ○ 0, Male (1)
- ○ 1, Female (2)
- ○ 2, Other (Specify) (3) ____

age
6. How old are you? (ENTER AGE IN YEARS: A NUMBER FROM 0 TO 99)

____

marital_status
7. What is your current marital status?

- ○ 1, Single (1)
- ○ 2, Partnered but not married (2)
- ○ 3, Married (3)
- ○ 4, Widowed (4)
- ○ 5, Divorced/Separated (5)

num_childrenn
8. How many children do you have? (ENTER A NUMBER FROM 0 TO 99)

____

Start of Block: Preparedness

gp
General perceptions:
These questions assess your personal feeling as a health care provider of being prepared or not to deal with the COVID-19 crises, as well as your perceptions of the situation in the facility you work in.

****Please answer all questions in this section to enable us to assess these issues completely**

---

gp_triage 1. How prepared are you as a health care provider in your ability to assess and triage patients with acute respiratory symptoms?

○ 0, Not at all prepared (1)

○ 1, A little prepared (2)

○ 2, Prepared (3)

○ 3, Very prepared (4)

○ 4, I don't know about this (5)

○ 5, Not applicable to my role (6)

---

gp_diagnose 2. How prepared are you as a health care provider in your ability to diagnose a patient with COVID-19?

○ 0, Not at all prepared (1)

○ 1, A little prepared (2)

○ 2, Prepared (3)

○ 3, Very prepared (4)

○ 4, I don't know about this (5)

○ 5, Not applicable to my role (6)

---

gp_manage 3. How prepared are you as a health care provider in your ability to manage a patient with diagnosed COVID-19?

- ◯ 0, Not at all prepared (1)
- ◯ 1, A little prepared (2)
- ◯ 2, Prepared (3)
- ◯ 3, Very prepared (4)
- ◯ 4, I don't know about this (5)
- ◯ 5, Not applicable to my role (6)

---

gp_PPE_on 4. How prepared are you as a health care provider in your ability to accurately put on PPE?

- ◯ 0, Not at all prepared (1)
- ◯ 1, A little prepared (2)
- ◯ 2, Prepared (3)
- ◯ 3, Very prepared (4)
- ◯ 4, I don't know about this (5)

---

gp_PPE_of 5. How prepared are you as a health care provider in your ability to safely take off PPE?

- ◯ 0, Not at all prepared (1)
- ◯ 1, A little prepared (2)
- ◯ 2, Prepared (3)
- ◯ 3, Very prepared (4)
- ◯ 4, I don't know about this (5)

---

gp_contact_precaut 6. How prepared are you as a health care provider in your ability to implement standard contact precautions?

- 0, Not at all prepared (1)
- 1, A little prepared (2)
- 2, Prepared (3)
- 3, Very prepared (4)
- 4, I don't know about this (5)

---

gp_airborne_precaut 7. How prepared are you as a health care provider in your ability to implement standard airborne precautions?

- 0, Not at all prepared (1)
- 1, A little prepared (2)
- 2, Prepared (3)
- 3, Very prepared (4)
- 4, I don't know about this (5)

---

gp_communicate 8. How prepared are you as a health care provider in your ability to communicate COVID-19 risks to your patients?

- 0, Not at all prepared (1)
- 1, A little prepared (2)
- 2, Prepared (3)
- 3, Very prepared (4)
- 4, I don't know about this (5)

---

gp_educate 9. How prepared are you as a health care provider in your ability to educate the public about COVID-19?

- 0, Not at all prepared (1)
- 1, A little prepared (2)
- 2, Prepared (3)
- 3, Very prepared (4)
- 4, I don't know about this (5)

---

gp_ration 10. How prepared are you as a health care provider in your ability to ration scarce life-saving commodities?

- 0, Not at all prepared (1)
- 1, A little prepared (2)
- 2, Prepared (3)
- 3, Very prepared (4)
- 4, I don't know about this (5)

---

gp_mental 11. As a health care provider, how mentally prepared are you to attend to a person diagnosed with COVID-19?

- 0, Not at all prepared (1)
- 1, A little prepared (2)
- 2, Prepared (3)
- 3, Very prepared (4)
- 4, I don't know about this (5)

---

gp_hf_diagnose 12. How prepared is the <u>health facility you work in to diagnose</u> COVID-19?

- ○ 0, Not at all prepared (1)
- ○ 1, A little prepared (2)
- ○ 2, Prepared (3)
- ○ 3, Very prepared (4)
- ○ 4, I don't know about this (5)

---

gp_hf_manage 13. How prepared is the <u>health facility you work in to manage</u> patients diagnosed with COVID-19?

- ○ 0, Not at all prepared (1)
- ○ 1, A little prepared (2)
- ○ 2, Prepared (3)
- ○ 3, Very prepared (4)
- ○ 4, I don't know about this (5)

---

gp_hf_spread_patient 14. How prepared is the health facility you work in to prevent the spread of COVID-19 to other patients and health workers if you had <u>an infected patient</u>?

- ○ 0, Not at all prepared (1)
- ○ 1, A little prepared (2)
- ○ 2, Prepared (3)
- ○ 3, Very prepared (4)
- ○ 4, I don't know about this (5)

---

gp_hf_spread_hw 15. How prepared is the health facility you work in to prevent spread of COVID-19 to other patients and health workers if you had an infected health worker?

- 0, Not at all prepared (1)
- 1, A little prepared (2)
- 2, Prepared (3)
- 3, Very prepared (4)
- 4, I don't know about this (5)

---

mngmt_communication 16. How will you describe communication from management of your facility or your in-charge/supervisor regarding the COVID-19 situation in your facility?

- 0, Very poor communication (1)
- 1, Poor communication (12)
- 2, Good communication (13)
- 3, Very good communication (15)

---

appreciate_managemnt 17. To what extent do you feel the management of your facility/in-charge/supervisor is appreciative of your work?

- 0, Not at all appreciative (1)
- 1, Somewhat appreciative (2)
- 2, Appreciative (3)
- 3, Very appreciative (4)

---

support_family 32. Is your family supportive of your work at this time of COVID-19?

- 0, Not at all supportive (1)
- 1, A little supportive (2)
- 2, Supportive (3)
- 3, Very supportive (4)

---

job_satisfied_prior
35. In general, how satisfied were you with your job before the COVID-19 crises?

- 0, Very dissatisfied (1)
- 1, Dissatisfied (2)
- 2, Satisfied (4)
- 3, Very satisfied (5)

---

job_satisfied_now
36. In general, how satisfied are you with your job now?

- 0, Very dissatisfied (1)
- 1, Dissatisfied (2)
- 2, Satisfied (4)
- 3, Very satisfied (5)

Start of Block: Stress/Burnout

stress_intro **These questions are to assess how the current situation may be affecting you.**

STRESS SCALE

The following questions ask about your feelings and thoughts during THE PAST MONTH.

In each question, you will be asked HOW OFTEN you felt or thought a certain way. Although some of the questions are similar, there are small differences between them and you should treat each one as a separate question. The best approach is to answer fairly quickly. That is, don't try to count up the exact number of times you felt a particular way, but respond based on what seems the best in general.

**Please answer all questions so we can accurately measure your levels of stress.

---

s1_upset 1. In the past month, how often have you been upset because of something that happened unexpectedly?

- ○ 0, Never (1)
- ○ 1, Almost Never (2)
- ○ 2, Sometimes (3)
- ○ 3, Fairly Often (4)
- ○ 4, Very Often (5)

---

s2_control_life 2. In the past month, how often have you felt unable to control the important things in your life?

- ○ 0, Never (1)
- ○ 1, Almost Never (2)
- ○ 2, Sometimes (3)
- ○ 3, Fairly Often (4)
- ○ 4, Very Often (5)

---

s3_nervous_stressed 3. In the past month, how often have you felt nervous or stressed?

- 0, Never (1)
- 1, Almost Never (2)
- 2, Sometimes (3)
- 3, Fairly Often (4)
- 4, Very Often (5)

---

s4_personal_problems 4. In the past month, how often have you felt confident about your ability to handle personal problems?

- 0, Never (1)
- 1, Almost Never (2)
- 2, Sometimes (3)
- 3, Fairly Often (4)
- 4, Very Often (5)

---

s5_going_your_way 5. In the past month, how often have you felt that things were going your way?

- 0, Never (1)
- 1, Almost Never (2)
- 2, Sometimes (3)
- 3, Fairly Often (4)
- 4, Very Often (5)

---

s6_not_cope 6. In the past month, how often have you found that you could not cope with all the things you had to do?

- 0, Never (1)
- 1, Almost Never (2)
- 2, Sometimes (3)
- 3, Fairly Often (4)
- 4, Very Often (5)

---

s7_control_irritatio 7. In the past month, how often have you been able to control irritations in your life?

- 0, Never (1)
- 1, Almost Never (2)
- 2, Sometimes (3)
- 3, Fairly Often (4)
- 4, Very Often (5)

---

s8_on_top_things 8. In the past month, how often have you felt that you were on top of things?

- 0, Never (1)
- 1, Almost Never (2)
- 2, Sometimes (3)
- 3, Fairly Often (4)
- 4, Very Often (5)

---

s9_angry_control 9. In the past month, how often have you been angry because of things that happened that were outside of your control?

- 0, Never (1)
- 1, Almost Never (2)
- 2, Sometimes (3)
- 3, Fairly Often (4)
- 4, Very Often (5)

---

s10_difficult_piling 10. In the past month, how often have you felt that difficulties were piling up so high that you could not overcome them?

- 0, Never (1)
- 1, Almost Never (2)
- 2, Sometimes (3)
- 3, Fairly Often (4)
- 4, Very Often (5)

---

burnout_intro
BURNOUT SCALE

**Please answer all questions so we can accurately measure your levels of burnout.

The following statements describe different feelings that you may feel at work. Please indicate how often, in the past 30 workdays, you have felt each of the following feelings:

---

bp1_tired 1. I feel tired

- 1, Never or almost never (1)
- 2, Very infrequently (2)
- 3, Quite infrequently (3)
- 4, Sometimes (4)
- 5, Quite frequently (5)
- 6, Very frequently (6)
- 7, Always or almost always (7)

---

bp2_no_energy 2. I have no energy for going to work in the morning

- 1, Never or almost never (1)
- 2, Very infrequently (2)
- 3, Quite infrequently (3)
- 4, Sometimes (4)
- 5, Quite frequently (5)
- 6, Very frequently (6)
- 7, Always or almost always (7)

---

bp3_drained 3. I feel physically drained

- 1, Never or almost never (1)
- 2, Very infrequently (2)
- 3, Quite infrequently (3)
- 4, Sometimes (4)
- 5, Quite frequently (5)
- 6, Very frequently (6)
- 7, Always or almost always (7)

---

bp4_fed_up 4. I feel fed up

- 1, Never or almost never (1)
- 2, Very infrequently (2)
- 3, Quite infrequently (3)
- 4, Sometimes (4)
- 5, Quite frequently (5)
- 6, Very frequently (6)
- 7, Always or almost always (7)

---

bp5_batteries_dead 5. I feel like my "batteries" are "dead"

- 1, Never or almost never (1)
- 2, Very infrequently (2)
- 3, Quite infrequently (3)
- 4, Sometimes (4)
- 5, Quite frequently (5)
- 6, Very frequently (6)
- 7, Always or almost always (7)

---

bp6_burned_out 6. I feel burned out

- 1, Never or almost never (1)
- 2, Very infrequently (2)
- 3, Quite infrequently (3)
- 4, Sometimes (4)
- 5, Quite frequently (5)
- 6, Very frequently (6)
- 7, Always or almost always (7)

---

bc7_slow_thinking 7. My thinking process is slow

- 1, Never or almost never (1)
- 2, Very infrequently (2)
- 3, Quite infrequently (3)
- 4, Sometimes (4)
- 5, Quite frequently (5)
- 6, Very frequently (6)
- 7, 7 Always or almost always (7)

---

bc8_concentrating 8. I have difficulty concentrating

- 1, Never or almost never (1)
- 2, Very infrequently (2)
- 3, Quite infrequently (3)
- 4, Sometimes (4)
- 5, Quite frequently (5)
- 6, Very frequently (6)
- 7, Always or almost always (7)

---

bc9_think_clearly 9. I feel I'm not thinking clearly

- 1, Never or almost never (1)
- 2, Very infrequently (2)
- 3, Quite infrequently (3)
- 4, Sometimes (4)
- 5, Quite frequently (5)
- 6, Very frequently (6)
- 7, Always or almost always (7)

---

bc10_not_focused 10. I feel I'm not focused in my thinking

- 1, Never or almost never (1)
- 2, Very infrequently (2)
- 3, Quite infrequently (3)
- 4, Sometimes (4)
- 5, Quite frequently (5)
- 6, Very frequently (6)
- 7, Always or almost always (7)

---

bc11_complex 11. I have difficulty thinking about complex things

- 1, Never or almost never (1)
- 2, Very infrequently (2)
- 3, Quite infrequently (3)
- 4, Sometimes (4)
- 5, Quite frequently (5)
- 6, Very frequently (6)
- 7, Always or almost always (7)

---

be12_sensitive_needs 12. I feel I am unable to be sensitive to the needs of coworkers and patients

- 1, Never or almost never (1)
- 2, Very infrequently (2)
- 3, Quite infrequently (3)
- 4, Sometimes (4)
- 5, Quite frequently (5)
- 6, Very frequently (6)
- 7, Always or almost always (7)

---

be13_emotion_invest 13. I feel I am not capable of investing emotionally in coworkers and patients

- 1, Never or almost never (1)
- 2, Very infrequently (2)
- 3, Quite infrequently (3)
- 4, Sometimes (4)
- 5, Quite frequently (5)
- 6, Very frequently (6)
- 7, Always or almost always (7)

---

be14_not_sympathetic 14. I feel I am not capable of being sympathetic to co-workers and patients

- 1, Never or almost never (1)
- 2, Very infrequently (2)
- 3, Quite infrequently (3)
- 4, Sometimes (4)
- 5, Quite frequently (5)
- 6, Very frequently (6)
- 7, Always or almost always (7)

End of Block: Stress/Burnout

Start of Block: COVID-19 Exposure

---

e4_manage_patient 4. Have you managed any patient confirmed to have COVID-19?

- 0, No (1)
- 1, Yes (2)
- 2, Don't know (4)
