# Supplementary material for: Job satisfaction among healthcare workers in Ghana and Kenya during the COVID-19 pandemic: Role of perceived preparedness, stress, and burnout
Source: PLOS Glob Public Health. 2021 Oct 13;1(10):e0000022. doi: 10.1371/journal.pgph.0000022 (PMC10021773; doi:10.1371/journal.pgph.0000022)
Supplement: S1 Table — (DOCX) [file pgph.0000022.s003.docx]

**S3: All Supplementary Tables**

**Table A: Difference in satisfaction, preparedness, stress, burnout at phase 1 and 2 and Kenya**

|  | Ghana Phase 1 | Ghana Phase 2 | Kenya |
| --- | --- | --- | --- |
| n | **476** | **408** | **128** |
| **Current job satisfaction**; N=1,012 \| χ (6) = 24.41, p < 0.0001 | % | % | % |
| Very dissatisfied | 8.82 | 4.17 | 12.5 |
| Dissatisfied | 34.66 | 28.68 | 22.66 |
| Satisfied | 49.16 | 54.9 | 55.47 |
| Very satisfied | 7.35 | 12.25 | 9.38 |
| **POR [95% CI]** | **0.609*****  **[0.472,0.785]** | **Ref.** | **0.778**  **[0.529,1.145]** |
|  |  |  |  |
| n | 476 | 408 | 128 |
| **Perceived preparedness**; N=1,012 \| χ (4) = 53.02, p < 0.0001 | % | % | % |
| Not prepared | 15.34 | 5.64 | 10.16 |
| A little prepared | 56.93 | 44.85 | 51.56 |
| Prepared | 27.73 | 49.51 | 38.28 |
| **POR [95% CI]** | **0.380*****  **[0.292,0.494]** | **Ref.** | **0.617***  **[0.420,0.907]** |
|  |  |  |  |
| n | 418 | 366 | 115 |
| **Perceived stress**; N=899 \| χ (4) = 12.81, p= 0.012 | % | % | % |
| Low stress | 31.1 | 30.33 | 20.87 |
| Moderate stress | 64.59 | 65.3 | 67.83 |
| High stress | 4.31 | 4.37 | 11.3 |
| **POR [95% CI]** | **0.967**  **[0.725,1.292]** | **Ref.** | **1.924****  **[1.209,3.064]** |
|  |  |  |  |
| n | 412 | 367 | 110 |
| **Perceived burnout**; N=889 \| χ (4) = 15.59, p= 0.004 | % | % | % |
| No burnout | 33.25 | 29.7 | 23.64 |
| Low burnout | 46.84 | 47.68 | 39.09 |
| High burnout | 19.9 | 22.62 | 37.27 |
| **POR [95% CI]** | **0.851**  **[0.655,1.107]** | **Ref.** | **1.732****  **[1.152,2.602]** |
|  |  |  |  |
| Exponentiated coefficients; 95% confidence intervals in brackets  * p<0.05, ** p<0.01, *** p<0.001 | | | |

**Table B: Multivariable model showing the association between Perceived preparedness and current job satisfaction, controlling for perceived burnout, stress, and other sociodemographic factors (except the job satisfaction prior to the pandemic variable).**

|  |  |  |  |
| --- | --- | --- | --- |
|  | Model I | Model II | Model III |
| *Independent variable* | IV: Perceived Preparedness | IV: Perceived  Preparedness & Burnout | IV: Perceived  Preparedness & Stress |
|  | APOR [95% CI] | APOR [95% CI] | APOR [95% CI] |
| **Perceived preparedness** |  |  |  |
| Not at all prepared | Ref. | Ref. | Ref. |
| A little prepared | 1.938**  [1.249,3.006] | 1.748*  [1.067,2.865] | 1.735*  [1.065,2.827] |
| Prepared | 3.716***  [2.236,6.176] | 3.646***  [2.058,6.459] | 3.730***  [2.119,6.565] |
| **Perceived burnout** |  |  |  |
| No burnout |  | Ref. |  |
| Low burnout |  | 0.709*  [0.512,0.982] |  |
| High burnout |  | 0.393***  [0.263,0.589] |  |
| **Perceived stress** |  |  |  |
| Low stress |  |  | Ref. |
| Moderate stress |  |  | 0.499***  [0.361,0.690] |
| High stress |  |  | 0.167***  [0.0837,0.335] |
|  |  |  |  |
| *control variable* |  |  |  |
| **Perceived management appreciation** |  |  |  |
| Not at all appreciative | Ref. | Ref. | Ref. |
| somewhat appreciative | 3.086***  [1.981,4.808] | 2.593***  [1.606,4.188] | 3.215***  [2.006,5.153] |
| Appreciative | 7.525***  [4.559,12.42] | 6.193***  [3.579,10.72] | 7.309***  [4.252,12.56] |
| Very appreciative | 9.301***  [4.832,17.90] | 7.755***  [3.857,15.59] | 9.322***  [4.656,18.67] |
| **Fear of infection** |  |  |  |
| Not fearful | Ref. | Ref. | Ref. |
| A little fearful | 0.714  [0.490,1.039] | 0.808  [0.541,1.208] | 0.909  [0.608,1.359] |
| Fearful | 0.619*  [0.408,0.939] | 0.686  [0.439,1.072] | 0.727  [0.465,1.136] |
| Very fearful | 0.315***  [0.199,0.499] | 0.382***  [0.233,0.629] | 0.429***  [0.262,0.702] |
| **Management communication** |  |  |  |
| Very poor | Ref. | Ref. | Ref. |
| Poor | 2.048**  [1.278,3.283] | 2.442***  [1.461,4.082] | 2.003**  [1.204,3.330] |
| Good | 2.228**  [1.354,3.667] | 2.691***  [1.561,4.641] | 2.206**  [1.289,3.776] |
| Very good | 2.228*  [1.101,4.510] | 2.401*  [1.118,5.155] | 2.158*  [1.012,4.604] |
| **Support from family** |  |  |  |
| Not at all supportive | Ref. | Ref. | Ref. |
| A little supportive | 0.960  [0.531,1.734] | 0.896  [0.466,1.724] | 0.722  [0.373,1.397] |
| Supportive | 1.627  [0.917,2.886] | 1.349  [0.713,2.552] | 1.095  [0.576,2.082] |
| Very supportive | 2.060*  [1.130,3.756] | 1.633  [0.839,3.176] | 1.329  [0.681,2.595] |
| **Gender** |  |  |  |
| Male | Ref. | Ref. | Ref. |
| Female | 1.079  [0.832,1.400] | 1.108  [0.834,1.474] | 1.057  [0.797,1.400] |
| **Age** |  |  |  |
| < 30 | Ref. | Ref. | Ref. |
| 30-39 | 0.949  [0.689,1.308] | 0.908  [0.643,1.283] | 0.903  [0.640,1.274] |
| 40+ | 0.966  [0.643,1.452] | 1.024  [0.657,1.596] | 0.939  [0.605,1.458] |
| **Marital status** |  |  |  |
| Single | Ref. | Ref. | Ref. |
| Currently married | 1.013  [0.738,1.389] | 0.859  [0.611,1.209] | 0.955  [0.679,1.342] |
| Formerly married (divorced/widowed) | 0.675  [0.301,1.515] | 0.469  [0.203,1.086] | 0.612  [0.263,1.425] |
| **Position** |  |  |  |
| Doctor | Ref. | Ref. | Ref. |
| Nurse/related | 1.011  [0.718,1.422] | 0.979  [0.677,1.417] | 1.033  [0.717,1.489] |
| Other | 1.273  [0.839,1.930] | 1.203  [0.769,1.882] | 1.263  [0.811,1.968] |
| **Health facility type** |  |  |  |
| Teaching hospital | Ref. | Ref. | Ref. |
| Other government facility | 0.907  [0.657,1.253] | 0.874  [0.619,1.236] | 0.943  [0.668,1.331] |
| Private/Missions | 0.914  [0.589,1.421] | 1.046  [0.652,1.678] | 1.044  [0.652,1.673] |
| **country/phases** |  |  |  |
| Ghana Phase 1 | 0.869  [0.658,1.147] | 0.895  [0.661,1.211] | 0.891  [0.658,1.205] |
| Ghana Phase 2 | Ref. | Ref. | Ref. |
| Kenya | 0.899  [0.585,1.383] | 1.134  [0.710,1.814] | 1.058  [0.665,1.685] |
| Pseudo R-squared (excluded prior job satisfaction from the model) | 0.154 | 0.168 | 0.175 |
| Sample size | 1009 | 880 | 890 |
| Exponentiated coefficients; 95% confidence intervals in brackets  * p<0.05, ** p<0.01, *** p<0.001 | | | |

**Table C: Mediation by stress and burnout in the association between perceived preparedness and job satisfaction**

|  | A little prepared | Prepared |
| --- | --- | --- |
|  | β [95% CI) | β [95% CI) |
| **Stress** |  |  |
| Total effect | 0.500 [-0.093 1.094] | 1.095*** [0.4296805, 1.76066] |
| Direct effect | 0.418 [-0.1739905, 1.010547] | 0.998** [0.3348093, 1.661975] |
| Indirect effect | 0.082 [-0.0483744, 0.2121921] | 0.097 [-0.0355113, 0.2290676] |
| % mediated | 16.38 | 8.84 |
| **Burnout** |  |  |
| Total effect | 0.509 [-.0957911, 1.113801] | 1.104*** [0.4238272, 1.783177] |
| Direct effect | 0.395 [-.2117013, 1.002421] | 0.963*** [0.2787749, 1.648116] |
| Indirect effect | 0.114 [-.022935, 0.2502259] | 0.140 [-0.0012386, 0.2813515] |
| % mediated | 22.3 | 12.69 |

**Table D:** **Multivariable model showing the association between Perceived preparedness and current job satisfaction, controlling for perceived burnout, stress, and other sociodemographic factors STRATIFIED BY GHANA in PHASES—Phase 1/Phase 2.**

|  | **Ghana Phase 1** | | | **Ghana Phase 2** | | |
| --- | --- | --- | --- | --- | --- | --- |
|  | Model I | Model II | Model III | Model I | Model II | Model III |
| *Independent variable* | IV: Perceived Preparedness | IV: Perceived Preparedness & Burnout | IV: Perceived Preparedness & Stress | IV: Perceived Preparedness | IV: Perceived Preparedness & Burnout | IV: Perceived Preparedness & Stress |
|  | APOR [95% CI] | APOR [95% CI] | APOR [95% CI] | APOR [95% CI] | APOR [95% CI] | APOR [95% CI] |
| **Perceived preparedness** |  |  |  |  |  |  |
| Not at all prepared | Ref. | Ref. | Ref. | Ref. | Ref. | Ref. |
| A little prepared | 1.531  [0.842,2.786] | 1.344  [0.685,2.638] | 1.422  [0.733,2.757] | 1.977  [0.711,5.501] | 1.236  [0.379,4.029] | 0.983  [0.301,3.217] |
| Prepared | 2.239*  [1.072,4.677] | 2.202  [0.959,5.052] | 2.371*  [1.046,5.373] | 3.310*  [1.128,9.710] | 1.977  [0.578,6.764] | 1.724  [0.504,5.897] |
| **Perceived burnout** |  |  |  |  |  |  |
| No burnout |  | Ref. |  |  | Ref. |  |
| Low burnout |  | 0.793  [0.481,1.308] |  |  | 0.604  [0.351,1.037] |  |
| High burnout |  | 0.464*  [0.239,0.902] |  |  | 0.427*  [0.215,0.848] |  |
| **Perceived stress** |  |  |  |  |  |  |
| Low stress |  |  | Ref. |  |  | Ref. |
| Moderate stress |  |  | 0.621*  [0.386,0.999] |  |  | 0.389**  [0.222,0.684] |
| High stress |  |  | 0.160**  [0.0448,0.575] |  |  | 0.167**  [0.0493,0.568] |
|  |  |  |  |  |  |  |
| *control variable* |  |  |  |  |  |  |
| **Job satisfaction prior to pandemic** |  |  |  |  |  |  |
| Very dissatisfied | Ref. | Ref. | Ref. | Ref. | Ref. | Ref. |
| dissatisfied | 4.789  [0.907,25.28] | 4.945  [0.752,32.52] | 4.407  [0.638,30.42] | 4.018  [0.856,18.87] | 3.834  [0.824,17.83] | 3.901  [0.841,18.10] |
| satisfied | 26.90***  [5.281,137.0] | 35.40***  [5.635,222.3] | 25.32***  [3.822,167.7] | 50.60***  [10.65,240.3] | 52.22***  [11.05,246.8] | 53.91***  [11.55,251.6] |
| very satisfied | 95.08***  [17.87,505.9] | 116.9***  [17.62,775.4] | 86.16***  [12.37,600.2] | 244.5***  [47.33,1263.5] | 285.7***  [54.23,1505.1] | 297.7***  [57.06,1552.8] |
| **Perceived management appreciation** |  |  |  |  |  |  |
| Not at all appreciative | Ref. | Ref. | Ref. | Ref. | Ref. | Ref. |
| somewhat appreciative | 3.472***  [1.805,6.681] | 2.830**  [1.385,5.784] | 3.659***  [1.821,7.354] | 1.520  [0.671,3.443] | 1.531  [0.639,3.671] | 1.608  [0.663,3.900] |
| Appreciative | 4.229***  [2.036,8.784] | 3.260**  [1.436,7.402] | 4.006***  [1.800,8.917] | 4.137**  [1.636,10.46] | 4.101**  [1.512,11.12] | 3.727*  [1.355,10.25] |
| Very appreciative | 2.683  [0.949,7.580] | 1.869  [0.591,5.907] | 2.156  [0.685,6.786] | 5.622**  [1.796,17.60] | 5.451**  [1.633,18.20] | 5.518**  [1.644,18.52] |
| **Fear of infection** |  |  |  |  |  |  |
| Not fearful | Ref. | Ref. | Ref. | Ref. | Ref. | Ref. |
| A little fearful | 0.558  [0.297,1.049] | 0.586  [0.293,1.172] | 0.618  [0.311,1.228] | 0.568  [0.323,1.000] | 0.628  [0.343,1.150] | 0.793  [0.429,1.468] |
| Fearful | 0.515  [0.260,1.018] | 0.546  [0.257,1.159] | 0.537  [0.255,1.132] | 0.484*  [0.248,0.945] | 0.484*  [0.239,0.978] | 0.574  [0.281,1.175] |
| Very fearful | 0.125***  [0.0589,0.267] | 0.145***  [0.0630,0.335] | 0.158***  [0.0694,0.358] | 0.335**  [0.153,0.736] | 0.336*  [0.146,0.777] | 0.409*  [0.177,0.943] |
| **Management communication** |  |  |  |  |  |  |
| Very poor | Ref. | Ref. | Ref. | Ref. | Ref. | Ref. |
| Poor | 1.647  [0.835,3.250] | 2.477*  [1.153,5.324] | 1.728  [0.816,3.662] | 2.500*  [1.027,6.086] | 3.233*  [1.266,8.255] | 3.260*  [1.266,8.398] |
| Good | 2.130*  [1.018,4.458] | 3.115**  [1.338,7.249] | 2.434*  [1.063,5.572] | 2.727*  [1.078,6.895] | 3.667**  [1.395,9.642] | 3.409*  [1.295,8.975] |
| Very good | 3.469*  [1.189,10.12] | 4.351*  [1.327,14.27] | 3.739*  [1.160,12.06] | 1.477  [0.426,5.114] | 1.366  [0.356,5.239] | 1.445  [0.378,5.529] |
| **Support from family** |  |  |  |  |  |  |
| Not at all supportive | Ref. | Ref. | Ref. | Ref. | Ref. | Ref. |
| A little supportive | 1.743  [0.716,4.247] | 1.649  [0.601,4.529] | 1.112  [0.393,3.149] | 0.631  [0.228,1.749] | 0.495  [0.168,1.457] | 0.625  [0.203,1.929] |
| Supportive | 2.266  [0.946,5.428] | 2.097  [0.774,5.680] | 1.351  [0.479,3.811] | 1.483  [0.555,3.967] | 1.111  [0.391,3.158] | 1.465  [0.493,4.354] |
| Very supportive | 2.535*  [1.016,6.322] | 2.236  [0.798,6.267] | 1.464  [0.504,4.255] | 1.844  [0.659,5.158] | 1.325  [0.443,3.964] | 1.745  [0.562,5.420] |
| **Gender** |  |  |  |  |  |  |
| Male | Ref. | Ref. | Ref. | Ref. | Ref. | Ref. |
| Female | 0.911  [0.605,1.372] | 0.842  [0.534,1.327] | 0.815  [0.522,1.272] | 1.163  [0.748,1.807] | 1.230  [0.760,1.989] | 1.193  [0.738,1.930] |
| **Age** |  |  |  |  |  |  |
| < 30 | Ref. | Ref. | Ref. | Ref. | Ref. | Ref. |
| 30-39 | 1.221  [0.759,1.966] | 1.069  [0.634,1.801] | 1.095  [0.652,1.839] | 1.159  [0.657,2.044] | 1.106  [0.607,2.016] | 1.163  [0.635,2.129] |
| 40+ | 1.196  [0.615,2.325] | 1.210  [0.571,2.564] | 1.154  [0.555,2.399] | 0.912  [0.454,1.833] | 0.898  [0.427,1.886] | 0.847  [0.403,1.780] |
| **Marital status** |  |  |  |  |  |  |
| Single | Ref. | Ref. | Ref. | Ref. | Ref. | Ref. |
| Currently married | 0.770  [0.474,1.249] | 0.688  [0.402,1.176] | 0.736  [0.432,1.257] | 0.741  [0.435,1.261] | 0.645  [0.368,1.131] | 0.642  [0.362,1.139] |
| Formerly married (divorced/widowed) | 0.680  [0.161,2.866] | 0.414  [0.0915,1.871] | 0.528  [0.111,2.506] | 1.066  [0.268,4.240] | 0.942  [0.234,3.784] | 0.879  [0.219,3.537] |
| **Position** |  |  |  |  |  |  |
| Doctor | Ref. | Ref. | Ref. | Ref. | Ref. | Ref. |
| Nurse/related | 1.367  [0.794,2.356] | 1.532  [0.845,2.780] | 1.488  [0.819,2.703] | 0.787  [0.446,1.387] | 0.863  [0.473,1.576] | 0.823  [0.450,1.504] |
| Other | 1.232  [0.617,2.460] | 1.249  [0.591,2.641] | 1.168  [0.556,2.455] | 1.258  [0.629,2.516] | 1.384  [0.664,2.887] | 1.296  [0.617,2.721] |
| **Health facility type** |  |  |  |  |  |  |
| Teaching hospital | Ref. | Ref. | Ref. | Ref. | Ref. | Ref. |
| Other government facility | 1.037  [0.624,1.723] | 1.184  [0.677,2.070] | 1.218  [0.700,2.121] | 0.831  [0.493,1.400] | 0.728  [0.417,1.273] | 0.850  [0.486,1.488] |
| Private/Missions | 1.314  [0.681,2.536] | 1.384  [0.677,2.829] | 1.543  [0.754,3.158] | 0.589  [0.262,1.326] | 0.691  [0.293,1.629] | 0.809  [0.341,1.923] |
| Pseudo R-squared | 0.243 | 0.260 | 0.258 | 0.289 | 0.308 | 0.319 |
| Sample size | 473 | 406 | 412 | 408 | 365 | 364 |
| Exponentiated coefficients; 95% confidence intervals in brackets  * p<0.05, ** p<0.01, *** p<0.001 | | | | | | |

**Table E:** **Multivariable model showing the association between Perceived preparedness and current job satisfaction, controlling for perceived burnout, stress, and other sociodemographic factors STRATIFIED BY COUNTRY—Ghana/Kenya.**

|  | **Ghana (Combined Phase 1 & 2 sample)** | | | **Kenya** | | |
| --- | --- | --- | --- | --- | --- | --- |
|  | Model I | Model II | Model III | Model I | Model II | Model III |
| *Independent variable* | IV: Perceived Preparedness | IV: Perceived Preparedness & Burnout | IV: Perceived Preparedness & Stress | IV: Perceived Preparedness | IV: Perceived Preparedness & Burnout | IV: Perceived Preparedness & Stress |
|  | APOR [95% CI] | APOR [95% CI] | APOR [95% CI] | APOR [95% CI] | APOR [95% CI] | APOR [95% CI] |
| **Perceived preparedness** |  |  |  |  |  |  |
| Not at all prepared | Ref. | Ref. | Ref. | Ref. | Ref. | Ref. |
| A little prepared | 1.738*  [1.050,2.875] | 1.543  [0.874,2.724] | 1.519  [0.865,2.667] | 1.796  [0.364,8.865] | 2.788  [0.385,20.21] | 3.055  [0.483,19.30] |
| Prepared | 2.968***  [1.679,5.248] | 2.739**  [1.447,5.186] | 2.916***  [1.548,5.493] | 5.146  [0.864,30.64] | 6.821  [0.774,60.08] | 7.604  [0.986,58.61] |
| **Perceived burnout** |  |  |  |  |  |  |
| No burnout |  | Ref. |  |  | Ref. |  |
| Low burnout |  | 0.767  [0.536,1.097] |  |  | 0.230*  [0.0594,0.893] |  |
| High burnout |  | 0.477**  [0.301,0.756] |  |  | 0.147**  [0.0349,0.617] |  |
| **Perceived stress** |  |  |  |  |  |  |
| Low stress |  |  | Ref. |  |  | Ref. |
| Moderate stress |  |  | 0.553**  [0.389,0.787] |  |  | 0.455  [0.138,1.501] |
| High stress |  |  | 0.195***  [0.0839,0.453] |  |  | 0.213  [0.0295,1.544] |
|  |  |  |  |  |  |  |
| *control variable* |  |  |  |  |  |  |
| **Job satisfaction prior to pandemic** |  |  |  |  |  |  |
| Very dissatisfied | Ref. | Ref. | Ref. | Ref. | Ref. | Ref. |
| dissatisfied | 4.162*  [1.398,12.39] | 3.954*  [1.244,12.57] | 3.890*  [1.223,12.37] | 0.00453***  [0.000186,0.110] | 0.000  [0.000,0.000] | 0.000  [0.000,0.000] |
| satisfied | 29.97***  [10.28,87.34] | 33.73***  [10.86,104.7] | 30.73***  [9.902,95.38] | 0.0608  [0.00337,1.095] | 0.000  [0.000,0.000] | 0.000  [0.000,0.000] |
| very satisfied | 116.7***  [38.30,355.6] | 130.1***  [39.79,425.1] | 121.2***  [37.05,396.2] | 0.281  [0.0169,4.686] | 0.000  [0.000,0.000] | 0.000  [0.000,0.000] |
| **Perceived management appreciation** |  |  |  |  |  |  |
| Not at all appreciative | Ref. | Ref. | Ref. | Ref. | Ref. | Ref. |
| somewhat appreciative | 2.378***  [1.452,3.896] | 2.216**  [1.301,3.775] | 2.592***  [1.530,4.393] | 8.181**  [1.776,37.68] | 2.129  [0.341,13.27] | 6.966*  [1.260,38.51] |
| Appreciative | 4.302***  [2.453,7.546] | 3.868***  [2.091,7.156] | 4.099***  [2.229,7.538] | 11.20**  [2.010,62.45] | 1.479  [0.176,12.45] | 9.026*  [1.377,59.17] |
| Very appreciative | 4.437***  [2.116,9.303] | 3.839***  [1.735,8.498] | 4.197***  [1.905,9.247] | 13.33*  [1.499,118.5] | 2.025  [0.143,28.77] | 9.921  [0.950,103.6] |
| **Fear of infection** |  |  |  |  |  |  |
| Not fearful | Ref. | Ref. | Ref. | Ref. | Ref. | Ref. |
| A little fearful | 0.556**  [0.371,0.832] | 0.595*  [0.386,0.915] | 0.667  [0.432,1.028] | 4.327  [0.806,23.25] | 2.647  [0.378,18.55] | 3.633  [0.527,25.04] |
| Fearful | 0.494**  [0.312,0.781] | 0.512**  [0.314,0.835] | 0.542*  [0.331,0.885] | 2.347  [0.509,10.82] | 1.562  [0.268,9.097] | 2.288  [0.394,13.30] |
| Very fearful | 0.175***  [0.104,0.295] | 0.192***  [0.109,0.337] | 0.216***  [0.124,0.377] | 1.187  [0.234,6.030] | 0.969  [0.148,6.325] | 1.426  [0.225,9.056] |
| **Management communication** |  |  |  |  |  |  |
| Very poor | Ref. | Ref. | Ref. | Ref. | Ref. | Ref. |
| Poor | 2.028**  [1.197,3.436] | 2.749***  [1.537,4.916] | 2.160**  [1.214,3.845] | 1.418  [0.243,8.281] | 2.486  [0.323,19.14] | 1.817  [0.219,15.11] |
| Good | 2.178**  [1.247,3.804] | 2.993***  [1.618,5.539] | 2.455**  [1.338,4.507] | 2.417  [0.385,15.16] | 8.551*  [1.041,70.25] | 3.161  [0.378,26.42] |
| Very good | 1.922  [0.881,4.193] | 2.126  [0.904,4.998] | 1.924  [0.824,4.494] | 3.864  [0.364,41.00] | 11.16  [0.808,154.2] | 4.386  [0.306,62.85] |
| **Support from family** |  |  |  |  |  |  |
| Not at all supportive | Ref. | Ref. | Ref. | Ref. | Ref. | Ref. |
| A little supportive | 1.140  [0.590,2.201] | 0.900  [0.438,1.847] | 0.830  [0.397,1.734] | 0.220  [0.00867,5.602] | 3.302  [0.0347,313.8] | 0.337  [0.0126,8.980] |
| Supportive | 1.910*  [1.007,3.621] | 1.421  [0.704,2.870] | 1.308  [0.637,2.687] | 0.812  [0.0369,17.89] | 5.756  [0.0607,545.8] | 0.746  [0.0316,17.62] |
| Very supportive | 2.208*  [1.131,4.311] | 1.569  [0.755,3.262] | 1.468  [0.697,3.093] | 0.940  [0.0430,20.52] | 3.772  [0.0401,354.6] | 0.597  [0.0259,13.74] |
| **Gender** |  |  |  |  |  |  |
| Male | Ref. | Ref. | Ref. | Ref. | Ref. | Ref. |
| Female | 1.018  [0.760,1.363] | 0.988  [0.718,1.360] | 0.950  [0.693,1.303] | 1.010  [0.412,2.477] | 1.098  [0.362,3.330] | 1.047  [0.388,2.822] |
| **Age** |  |  |  |  |  |  |
| < 30 | Ref. | Ref. | Ref. | Ref. | Ref. | Ref. |
| 30-39 | 1.207  [0.844,1.725] | 1.098  [0.747,1.613] | 1.141  [0.777,1.674] | 0.468  [0.139,1.580] | 0.578  [0.138,2.419] | 0.539  [0.136,2.144] |
| 40+ | 1.094  [0.685,1.746] | 1.101  [0.660,1.836] | 1.066  [0.643,1.766] | 0.674  [0.191,2.378] | 0.967  [0.228,4.100] | 0.928  [0.229,3.762] |
| **Marital status** |  |  |  |  |  |  |
| Single | Ref. | Ref. | Ref. | Ref. | Ref. | Ref. |
| Currently married | 0.770  [0.543,1.094] | 0.674*  [0.461,0.984] | 0.704  [0.482,1.029] | 0.938  [0.278,3.162] | 0.750  [0.185,3.033] | 0.923  [0.248,3.443] |
| Formerly married (divorced/widowed) | 0.867  [0.321,2.342] | 0.643  [0.230,1.800] | 0.697  [0.249,1.951] | 1.992  [0.246,16.10] | 0.804  [0.0718,9.004] | 1.131  [0.105,12.13] |
| **Position** |  |  |  |  |  |  |
| Doctor | Ref. | Ref. | Ref. | Ref. | Ref. | Ref. |
| Nurse/related | 1.027  [0.699,1.509] | 1.111  [0.736,1.678] | 1.071  [0.710,1.616] | 0.905  [0.295,2.770] | 0.573  [0.138,2.377] | 1.078  [0.295,3.937] |
| Other | 1.320  [0.816,2.134] | 1.358  [0.813,2.268] | 1.248  [0.748,2.082] | 1.019  [0.313,3.320] | 0.637  [0.149,2.734] | 1.044  [0.278,3.914] |
| **Health facility type** |  |  |  |  |  |  |
| Teaching hospital | Ref. | Ref. | Ref. | Ref. | Ref. | Ref. |
| Other government facility | 0.971  [0.681,1.385] | 0.989  [0.675,1.451] | 1.074  [0.733,1.572] | 1.108  [0.290,4.233] | 0.630  [0.137,2.903] | 0.714  [0.154,3.318] |
| Private/Missions | 0.891  [0.542,1.466] | 0.992  [0.581,1.691] | 1.090  [0.639,1.859] | 0.783  [0.158,3.888] | 0.830  [0.129,5.324] | 0.526  [0.0879,3.149] |
| Pseudo R-squared | 0.248 | 0.265 | 0.267 | 0.336 | 0.409 | 0.379 |
| Sample size | 881 | 771 | 776 | 128 | 109 | 114 |
| Exponentiated coefficients; 95% confidence intervals in brackets  * p<0.05, ** p<0.01, *** p<0.001 | | | | | | |

**Table F:** **Multivariable model showing the association between Perceived preparedness and current job satisfaction, controlling for perceived burnout, stress, and other sociodemographic factors (except the job satisfaction prior to the pandemic variable) STRATIFIED BY GHANA in PHASES—Phase 1/Phase 2.**

|  | **Ghana Phase 1** | | | **Ghana Phase 2** | | |
| --- | --- | --- | --- | --- | --- | --- |
|  | Model I | Model II | Model III | Model I | Model II | Model III |
| *Independent variable* | IV: Perceived Preparedness | IV: Perceived Preparedness & Burnout | IV: Perceived Preparedness & Stress | IV: Perceived Preparedness | IV: Perceived Preparedness & Burnout | IV: Perceived Preparedness & Stress |
|  | APOR [95% CI] | APOR [95% CI] | APOR [95% CI] | APOR [95% CI] | APOR [95% CI] | APOR [95% CI] |
| **Perceived preparedness** |  |  |  |  |  |  |
| Not at all prepared | Ref. | Ref. | Ref. | Ref. | Ref. | Ref. |
| A little prepared | 2.015*  [1.145,3.544] | 1.685  [0.894,3.178] | 1.738  [0.927,3.259] | 1.460  [0.565,3.773] | 1.156  [0.378,3.536] | 0.933  [0.302,2.876] |
| Prepared | 3.158**  [1.557,6.404] | 2.910**  [1.311,6.460] | 3.090**  [1.397,6.835] | 3.399*  [1.254,9.216] | 2.770  [0.865,8.872] | 2.406  [0.747,7.744] |
| **Perceived burnout** |  |  |  |  |  |  |
| No burnout |  | Ref. |  |  | Ref. |  |
| Low burnout |  | 0.634  [0.391,1.029] |  |  | 0.770  [0.464,1.279] |  |
| High burnout |  | 0.356**  [0.190,0.668] |  |  | 0.476*  [0.251,0.902] |  |
| **Perceived stress** |  |  |  |  |  |  |
| Low stress |  |  | Ref. |  |  | Ref. |
| Moderate stress |  |  | 0.579*  [0.364,0.920] |  |  | 0.403***  [0.238,0.684] |
| High stress |  |  | 0.115***  [0.0351,0.375] |  |  | 0.181**  [0.0577,0.570] |
|  |  |  |  |  |  |  |
| *control variable* |  |  |  |  |  |  |
| **Perceived management appreciation** |  |  |  |  |  |  |
| Not at all appreciative | Ref. | Ref. | Ref. | Ref. | Ref. | Ref. |
| somewhat appreciative | 3.896***  [2.083,7.288] | 3.033**  [1.537,5.987] | 3.921***  [2.015,7.629] | 1.566  [0.715,3.427] | 1.598  [0.689,3.709] | 1.643  [0.697,3.869] |
| Appreciative | 6.069***  [3.011,12.24] | 4.594***  [2.093,10.08] | 5.574***  [2.572,12.08] | 5.938***  [2.466,14.30] | 6.139***  [2.377,15.86] | 5.602***  [2.130,14.73] |
| Very appreciative | 4.477**  [1.644,12.19] | 3.677*  [1.229,11.00] | 4.302**  [1.435,12.89] | 11.14***  [3.811,32.58] | 11.71***  [3.783,36.24] | 12.01***  [3.832,37.65] |
| **Fear of infection** |  |  |  |  |  |  |
| Not fearful | Ref. | Ref. | Ref. | Ref. | Ref. | Ref. |
| A little fearful | 0.642  [0.348,1.185] | 0.762  [0.391,1.485] | 0.780  [0.401,1.518] | 0.672  [0.395,1.144] | 0.776  [0.442,1.363] | 0.993  [0.559,1.764] |
| Fearful | 0.586  [0.303,1.135] | 0.737  [0.358,1.516] | 0.690  [0.336,1.417] | 0.444*  [0.236,0.838] | 0.483*  [0.249,0.937] | 0.604  [0.308,1.186] |
| Very fearful | 0.202***  [0.0982,0.415] | 0.278**  [0.127,0.609] | 0.269***  [0.124,0.584] | 0.360**  [0.170,0.762] | 0.386*  [0.175,0.851] | 0.468  [0.211,1.037] |
| **Management communication** |  |  |  |  |  |  |
| Very poor | Ref. | Ref. | Ref. | Ref. | Ref. | Ref. |
| Poor | 2.087*  [1.098,3.968] | 2.876**  [1.401,5.903] | 2.023  [0.995,4.115] | 2.305  [0.990,5.367] | 2.317  [0.956,5.613] | 2.366  [0.968,5.783] |
| Good | 2.738**  [1.357,5.523] | 3.526**  [1.587,7.835] | 2.847**  [1.296,6.255] | 2.160  [0.904,5.163] | 2.261  [0.917,5.573] | 2.091  [0.848,5.157] |
| Very good | 3.940**  [1.407,11.03] | 5.027**  [1.617,15.63] | 4.355*  [1.420,13.36] | 0.923  [0.280,3.038] | 0.685  [0.192,2.446] | 0.724  [0.201,2.603] |
| **Support from family** |  |  |  |  |  |  |
| Not at all supportive | Ref. | Ref. | Ref. | Ref. | Ref. | Ref. |
| A little supportive | 1.318  [0.568,3.054] | 1.183  [0.457,3.064] | 0.807  [0.303,2.147] | 0.871  [0.337,2.250] | 0.780  [0.284,2.143] | 0.806  [0.283,2.296] |
| Supportive | 1.673  [0.734,3.810] | 1.421  [0.557,3.627] | 0.915  [0.345,2.424] | 1.938  [0.776,4.838] | 1.656  [0.622,4.408] | 1.823  [0.662,5.020] |
| Very supportive | 2.375  [1.000,5.639] | 1.948  [0.735,5.165] | 1.257  [0.458,3.452] | 2.356  [0.900,6.166] | 1.902  [0.678,5.341] | 2.048  [0.709,5.911] |
| **Gender** |  |  |  |  |  |  |
| Male | Ref. | Ref. | Ref. | Ref. | Ref. | Ref. |
| Female | 0.957  [0.645,1.419] | 0.898  [0.581,1.388] | 0.858  [0.558,1.321] | 1.468  [0.968,2.228] | 1.567  [0.997,2.461] | 1.553  [0.990,2.435] |
| **Age** |  |  |  |  |  |  |
| < 30 | Ref. | Ref. | Ref. | Ref. | Ref. | Ref. |
| 30-39 | 1.150  [0.730,1.811] | 1.059  [0.643,1.742] | 1.063  [0.647,1.747] | 0.831  [0.486,1.421] | 0.773  [0.439,1.361] | 0.821  [0.465,1.448] |
| 40+ | 1.002  [0.530,1.892] | 1.161  [0.565,2.385] | 1.042  [0.515,2.107] | 0.818  [0.420,1.594] | 0.774  [0.382,1.569] | 0.734  [0.362,1.487] |
| **Marital status** |  |  |  |  |  |  |
| Single | Ref. | Ref. | Ref. | Ref. | Ref. | Ref. |
| Currently married | 0.908  [0.572,1.443] | 0.808  [0.484,1.348] | 0.917  [0.551,1.526] | 0.942  [0.571,1.553] | 0.842  [0.499,1.421] | 0.837  [0.492,1.423] |
| Formerly married (divorced/widowed) | 1.005  [0.251,4.028] | 0.550  [0.128,2.354] | 0.768  [0.168,3.502] | 1.090  [0.284,4.181] | 1.001  [0.262,3.831] | 0.990  [0.262,3.734] |
| **Position** |  |  |  |  |  |  |
| Doctor | Ref. | Ref. | Ref. | Ref. | Ref. | Ref. |
| Nurse/related | 1.249  [0.741,2.105] | 1.415  [0.801,2.498] | 1.348  [0.762,2.381] | 0.649  [0.377,1.116] | 0.641  [0.362,1.135] | 0.632  [0.358,1.116] |
| Other | 1.143  [0.589,2.215] | 1.190  [0.578,2.450] | 1.101  [0.539,2.247] | 1.207  [0.618,2.358] | 1.250  [0.619,2.523] | 1.288  [0.633,2.618] |
| **Health facility type** |  |  |  |  |  |  |
| Teaching hospital | Ref. | Ref. | Ref. | Ref. | Ref. | Ref. |
| Other government facility | 1.023  [0.627,1.672] | 1.048  [0.613,1.790] | 1.152  [0.676,1.963] | 0.734  [0.448,1.203] | 0.675  [0.400,1.139] | 0.734  [0.434,1.241] |
| Private/Missions | 1.465  [0.776,2.765] | 1.586  [0.800,3.147] | 1.804  [0.904,3.598] | 0.643  [0.296,1.397] | 0.764  [0.339,1.726] | 0.826  [0.363,1.877] |
| Pseudo R-squared | 0.161 | 0.178 | 0.185 | 0.169 | 0.180 | 0.191 |
| Sample size | 473 | 406 | 412 | 408 | 365 | 364 |
| Exponentiated coefficients; 95% confidence intervals in brackets  * p<0.05, ** p<0.01, *** p<0.001 | | | | | | |

**Table G:** **Multivariable model showing the association between Perceived preparedness and current job satisfaction, controlling for perceived burnout, stress, and other sociodemographic factors (except the job satisfaction prior to the pandemic variable) STRATIFIED BY COUNTRY—Ghana/Kenya.**

|  | **Ghana** | | | **Kenya** | | |
| --- | --- | --- | --- | --- | --- | --- |
|  | Model I | Model II | Model III | Model I | Model II | Model III |
| *Independent variable* | IV: Perceived Preparedness | IV: Perceived Preparedness & Burnout | IV: Perceived Preparedness & Stress | IV: Perceived Preparedness | IV: Perceived Preparedness & Burnout | IV: Perceived Preparedness & Stress |
|  | APOR [95% CI] | APOR [95% CI] | APOR [95% CI] | APOR [95% CI] | APOR [95% CI] | APOR [95% CI] |
| **Perceived preparedness** |  |  |  |  |  |  |
| Not at all prepared | Ref. | Ref. | Ref. | Ref. | Ref. | Ref. |
| A little prepared | 2.016**  [1.257,3.232] | 1.778*  [1.046,3.020] | 1.702*  [1.006,2.881] | 3.141  [0.676,14.59] | 2.212  [0.385,12.72] | 3.528  [0.611,20.38] |
| Prepared | 3.920***  [2.289,6.715] | 3.697***  [2.026,6.744] | 3.823***  [2.101,6.955] | 6.273*  [1.134,34.70] | 4.593  [0.671,31.45] | 7.242*  [1.075,48.79] |
| **Perceived burnout** |  |  |  |  |  |  |
| No burnout |  | Ref. |  |  | Ref. |  |
| Low burnout |  | 0.738  [0.523,1.042] |  |  | 0.441  [0.136,1.430] |  |
| High burnout |  | 0.420***  [0.272,0.649] |  |  | 0.235*  [0.0673,0.820] |  |
| **Perceived stress** |  |  |  |  |  |  |
| Low stress |  |  | Ref. |  |  | Ref. |
| Moderate stress |  |  | 0.524***  [0.373,0.738] |  |  | 0.362  [0.120,1.089] |
| High stress |  |  | 0.172***  [0.0776,0.383] |  |  | 0.138*  [0.0227,0.840] |
|  |  |  |  |  |  |  |
| *control variable* |  |  |  |  |  |  |
| **Perceived management appreciation** |  |  |  |  |  |  |
| Not at all appreciative | Ref. | Ref. | Ref. | Ref. | Ref. | Ref. |
| somewhat appreciative | 2.494***  [1.550,4.011] | 2.236**  [1.340,3.730] | 2.635***  [1.586,4.376] | 11.77***  [2.776,49.89] | 7.303*  [1.299,41.06] | 10.53**  [2.141,51.79] |
| Appreciative | 6.126***  [3.577,10.49] | 5.537***  [3.068,9.994] | 5.921***  [3.295,10.64] | 29.89***  [6.115,146.1] | 15.42**  [2.467,96.44] | 24.14***  [4.388,132.9] |
| Very appreciative | 7.343***  [3.613,14.92] | 6.811***  [3.191,14.54] | 7.649***  [3.594,16.28] | 68.04***  [9.252,500.4] | 47.46***  [4.984,452.0] | 49.99***  [5.970,418.7] |
| **Fear of infection** |  |  |  |  |  |  |
| Not fearful | Ref. | Ref. | Ref. | Ref. | Ref. | Ref. |
| A little fearful | 0.617*  [0.417,0.912] | 0.718  [0.474,1.088] | 0.806  [0.531,1.224] | 3.289  [0.692,15.62] | 2.447  [0.411,14.57] | 3.530  [0.593,21.01] |
| Fearful | 0.519**  [0.333,0.809] | 0.605*  [0.378,0.968] | 0.647  [0.403,1.038] | 2.850  [0.658,12.35] | 1.895  [0.355,10.11] | 2.544  [0.492,13.16] |
| Very fearful | 0.247***  [0.150,0.405] | 0.305***  [0.179,0.520] | 0.325***  [0.192,0.552] | 1.570  [0.337,7.315] | 1.351  [0.234,7.795] | 2.131  [0.372,12.21] |
| **Management communication** |  |  |  |  |  |  |
| Very poor | Ref. | Ref. | Ref. | Ref. | Ref. | Ref. |
| Poor | 2.239**  [1.358,3.694] | 2.630***  [1.522,4.546] | 2.160**  [1.254,3.718] | 1.287  [0.226,7.342] | 2.459  [0.326,18.52] | 1.938  [0.261,14.41] |
| Good | 2.347**  [1.383,3.985] | 2.693***  [1.508,4.812] | 2.289**  [1.290,4.062] | 1.658  [0.274,10.02] | 4.448  [0.587,33.70] | 2.428  [0.326,18.10] |
| Very good | 1.851  [0.872,3.932] | 1.877  [0.829,4.250] | 1.759  [0.781,3.964] | 5.239  [0.523,52.46] | 12.93*  [1.030,162.4] | 6.850  [0.532,88.27] |
| **Support from family** |  |  |  |  |  |  |
| Not at all supportive | Ref. | Ref. | Ref. | Ref. | Ref. | Ref. |
| A little supportive | 1.081  [0.587,1.990] | 0.957  [0.489,1.870] | 0.811  [0.409,1.610] | 0.182  [0.00835,3.954] | 0.324  [0.00555,18.92] | 0.248  [0.0116,5.304] |
| Supportive | 1.738  [0.960,3.149] | 1.422  [0.737,2.743] | 1.200  [0.613,2.350] | 0.579  [0.0291,11.55] | 0.701  [0.0115,42.93] | 0.494  [0.0243,10.05] |
| Very supportive | 2.335**  [1.249,4.366] | 1.805  [0.907,3.594] | 1.530  [0.758,3.085] | 0.477  [0.0243,9.348] | 0.439  [0.00731,26.39] | 0.357  [0.0179,7.122] |
| **Gender** |  |  |  |  |  |  |
| Male | Ref. | Ref. | Ref. | Ref. | Ref. | Ref. |
| Female | 1.150  [0.869,1.522] | 1.142  [0.841,1.549] | 1.098  [0.811,1.487] | 1.000  [0.426,2.348] | 1.347  [0.500,3.630] | 1.107  [0.433,2.833] |
| **Age** |  |  |  |  |  |  |
| < 30 | Ref. | Ref. | Ref. | Ref. | Ref. | Ref. |
| 30-39 | 1.050  [0.747,1.476] | 0.971  [0.674,1.401] | 1.003  [0.696,1.446] | 0.543  [0.173,1.706] | 0.563  [0.157,2.026] | 0.519  [0.143,1.889] |
| 40+ | 0.954  [0.609,1.494] | 1.006  [0.616,1.643] | 0.945  [0.582,1.534] | 0.491  [0.143,1.688] | 0.597  [0.154,2.315] | 0.608  [0.157,2.355] |
| **Marital status** |  |  |  |  |  |  |
| Single | Ref. | Ref. | Ref. | Ref. | Ref. | Ref. |
| Currently married | 0.951  [0.681,1.326] | 0.854  [0.596,1.223] | 0.910  [0.635,1.303] | 0.935  [0.291,3.003] | 0.531  [0.145,1.942] | 0.853  [0.245,2.973] |
| Formerly married (divorced/widowed) | 1.023  [0.390,2.681] | 0.741  [0.277,1.986] | 0.844  [0.314,2.272] | 0.411  [0.0644,2.626] | 0.117  [0.0129,1.056] | 0.243  [0.0284,2.079] |
| **Position** |  |  |  |  |  |  |
| Doctor | Ref. | Ref. | Ref. | Ref. | Ref. | Ref. |
| Nurse/related | 0.923  [0.638,1.336] | 0.975  [0.658,1.446] | 0.941  [0.636,1.393] | 1.118  [0.399,3.136] | 0.760  [0.220,2.631] | 1.524  [0.476,4.876] |
| Other | 1.235  [0.778,1.962] | 1.281  [0.782,2.097] | 1.226  [0.749,2.005] | 1.277  [0.427,3.819] | 0.840  [0.228,3.099] | 1.572  [0.479,5.163] |
| **Health facility type** |  |  |  |  |  |  |
| Teaching hospital | Ref. | Ref. | Ref. | Ref. | Ref. | Ref. |
| Other government facility | 0.923  [0.657,1.298] | 0.897  [0.623,1.293] | 0.986  [0.684,1.420] | 0.912  [0.264,3.152] | 0.460  [0.110,1.924] | 0.575  [0.141,2.348] |
| Private/Missions | 1.008  [0.625,1.628] | 1.159  [0.695,1.932] | 1.248  [0.747,2.086] | 0.687  [0.161,2.928] | 0.364  [0.0663,2.001] | 0.425  [0.0819,2.201] |
| Pseudo R-squared | 0.152 | 0.165 | 0.172 | 0.250 | 0.265 | 0.281 |
| Sample size | 881 | 771 | 776 | 128 | 109 | 114 |
| Exponentiated coefficients; 95% confidence intervals in brackets  * p<0.05, ** p<0.01, *** p<0.001 | | | | | | |
