# Supplementary material for: Job satisfaction among healthcare workers in Ghana and Kenya during the COVID-19 pandemic: Role of perceived preparedness, stress, and burnout
Source: PLOS Glob Public Health. 2021 Oct 13;1(10):e0000022. doi: 10.1371/journal.pgph.0000022 (PMC10021773; doi:10.1371/journal.pgph.0000022)
Supplement: Attachment — Submitted filename: Response to reviewers-8-18-21.docx [file pgph.0000022.s004.docx]

| **COMMENTS** | **RESPONSE** | **Page no** |
| --- | --- | --- |
| **Additional Editor Comments (if provided):** |  |  |
| Thank you very much for submitting your manuscript to PLOS Global Health. The manuscript addresses an important area of research in global health and, as reviewer 1 highlighted, makes a novel contribution in this area of investigation. Reviewer 2 recommends some revisions, particularly incorporating some additional discussion of some of the findings and limitations of the study. Therefore, I invite you to respond to the reviewer’s comments and revise the manuscript. | Thank you. We are pleased that the reviewers found our research impactful and that it addresses an important area in global health. Below, and in corresponding pages of the manuscript, we outline the detailed responses and edits to the reviewers’ comments. |  |
| In discussing the settings of Ghana and Kenya (in the methods section), you might also briefly note if there are any key differences worth highlighting in addition to the similarities you discuss regarding Covid-19 trends and constrained health systems in the two countries. For instance, you indicate that there are nearly four times as many nurses/midwives per 10,000 population in Ghana as compared to Kenya (42 per 10,000 compared to 8.3 per 10,000); does this difference have any potential relevance for job satisfaction among healthcare workers in the two countries? Generally, as Reviewer 2 suggests, some additional explicit discussion of the rationale for comparing healthcare workers in Ghana and Kenya, including differences in methodology between the countries (differences in time frame, sample size, use of a raffle incentive in Ghana but not in Kenya, etc.), and some additional discussion of the potential limitations associated with these differences that might affect how the findings are interpreted, would further strengthen the manuscript. | We have addressed the reviewers’ comments regarding the differences and similarities in the two study settings. First, we provide the rationale for conducting the study in the two countries and highlight the differences in the HCW-patient ratio in the two countries under the settings section under methods. Second, we also include and provide a description of the supplemental tables with stratified results of both the different phases of the study, as well as the different settings i.e. Ghana and Kenya, in the results section. Finally, we discuss the significance of these differences in the discussion sections, including highlighting limitations | Settings- Page 6  Supplemental tables-S4 Table4a  S4 Table4b  S5 Table5a  S5 Table5b  Results- page 33  Methodology- page 6  Discussion- pages 28-30 |
|  |  |  |
| **Reviewer #1:** |  |  |
| I read the manuscript entitled “Job satisfaction among healthcare workers in Ghana and Kenya during the COVID-19 pandemic: role of perceived preparedness, stress, and burnout.” Overall, this is a clear, detailed, and well-written manuscript. The introduction is relevant, and theory based. Sufficient information about the previous study findings is presented for readers to follow the present study rationale and procedures. The methods are generally appropriate. Overall, the results are clear. The authors make a systematic contribution to the research literature in this area of investigation. Overall, this is a high-quality manuscript that has implications for the theoretical basis. Regards | Thank you very much for taking time to review our manuscript and for providing these positive comments. We are pleased that you found our work to be of significant contribution to the field, and that it advances theoretical understanding of job satisfaction among healthcare workers. | N/A |
|  |  |  |
| **Reviewer #2:** |  |  |
| Thank you for allowing me to review this interesting manuscript. Here are a few of my thoughts and suggestions: | We are appreciate your thoughtful comments. We have revised the manuscript accordingly and responded to the comments raised, in detail below. |  |
| I am unable to tell from the manuscript if there was difference between the different type of healthcare workers survey in both Ghana and Kenya. | As shown in table 1, although the types of healthcare providers participating in the survey in Ghana and Kenya were different, these differences were not substantial.  The proportion who were doctors were 20.2%, 25.5%, and 28.9% respectively for Ghana phase1, Ghana phase 2, and Kenya; 63.2%, 55.9%, and 44.5% respectively for nurses; and 16.6%, 18.6%, 26. 6% for allied health workers.  We have edited the sample description to highlight this. | Pages 12-13 |
| Who makes up the allied healthcare workers? | Allied healthcare workers are medical laboratory, pharmacy, public health, and other health staff not classified as doctors or nurses.  We have edited the first sentence of data collection section to include a description of this. | Page 8 |
| Were nurse more affected than physician or vice versa?  How physicians were in training as they tend to suffer more from psychological differences. | As shown in tables 2 and 3, there was no significant difference in satisfaction level by provider type. Further, we note this finding in the last sentences of the bivariable results section.  We acknowledge physicians in training may suffer from the psychological effects of both COVID-19 and their relative and perceived preparedness to manage the pandemic. However, we are unable to examine the effects among this group as we grouped residents and specialist doctors in the same category in the survey. We have noted this in the limitations section. | Page 16 and 32 |
| b. It is also unclear from the survey that all healthcare workers were involved with patient who had COVID-19. This would be useful to fully determine a solid relationship. | Unfortunately, the first phase of the survey did not include a question on whether providers were involved with a patient who had COVID. This question was added in the second phase of the survey based on lessons from the first phase of the study. In Ghana Phase 2, 354 participants responded to the question, and 37 % indicated that they were involved with managing covid19 patients. In Kenya, 100 responded to the question of which 60% reported they had been involved with management of a patient with COVID and the remaining responded they could not tell.  We excluded this question from the analysis because it was missing for all phase 1 respondents and many in phase 2 had uncertainty about it. But per your comments, we run supplemental analysis for those who responded to this question shown in S Table 6.  This analysis is limited to those who indicated Yes or No to the question. Those who said they could not tell were not added to the analyses given that this group is different from those who were sure of not dealing with covid19 patients. Therefore, all cases (60) in the Kenyan sample in these supplemental analyses had managed COVID-19 patients, and the variable cannot be included as a predictor in the model. For the Ghanaian sample, there is no statistically significant difference in current job satisfaction between health workers who managed COVID-19 patients and those who don’t. However, the samples on which these additional analyses were performed is rather too small to satisfactorily build a regression model that regressed all explanatory variables on job satisfaction, thus these results are not comparable to the rest of the results and can be confusing to readers. Also, given the large number of missing cases on this variable, it is difficult to make any solid conclusions. Thus we have only included this table for review and do not recommend it be included in the published paper.  It is however important to note that health workers in both countries were generally in some form of fear about dealing with clients because of covid-19 due to inadequate testing in both countries, and fear of covid19 has already been established in our analyses to be associated with current job satisfaction. | Additional file with Table S6 |
| c. The study was conducted by mainly OBGYN department, who I am not sure are fully involved in COVID care, certainly at hospital in Kenya. | We note that the study investigative teams’ primary affiliation may have been mistaken for the COVID-19 care location/setting. We would like to clarify that our multidisciplinary team members represent researchers, clinicians, and public health experts (Two of our investigative team are OBGYN’s). However, the survey was done with providers in Ghana and Kenya, many of whom were involved in the provision of COVID care. Further, our comprehensive survey distribution strategy, which utilized general HCW groups and professional associations, also ensured that the survey spanned different sub specialties, which is reflective of the broad provider types involved in COVID-19 response in Ghana and Kenya. | Page 8 |
| d. The offering of a raffle to the participants in Ghana would, I think, cause a bias in the responses. This should be a limitation. Alternatively, this population should be removed. | We acknowledge this have included it in the limitations section. However, we note that removing this will mean excluding all of the phase 2 Ghana sample, which we do not believe is the ideal approach given the wealth of data and its importance. We have, however, addressed this by conducting further analyses for the two phases and countries separately; these data are presented in the supplemental files S4 and S5. As shown, it does not change the interpretation of the results. | Page 32, 36 |
| e. One of the questions that always comes up time and time again is the validation of the survey tools. Are the surveys used in this study validated in West and East Africa? Can the authors provide justification to the validations process or quote other studies that have used these tools? | Yes, all survey tools have been validated in east and west Africa. We note this in the description of the measures with citations to relevant studies. | Page 9-10 |
| f. The authors did not really provide a justification or explanation about why symptoms were worse in the first wave rather than the second phase of the study. This would be key to help explain their findings. I would assume that as number of waves increase, the symptoms would worsen. | We note that the reviewer intended to highlight worse outcomes related to satisfaction, vis-à-vis “*symptoms*”, since our study did not examine symptoms. We have thus included a paragraph in the discussions section on why satisfaction was worse in the earlier phase of the pandemic, where we note that one of the primary reasons is largely related to the relative newness of COVID-19, and the uncertainty around transmission, limited guidelines for clinical management, and the lack of promising vaccines or treatments. | Page 28-29 |
| g. The number of participants in Kenya was only 128. This is a huge limitation and such low numbers cannot be generalized to the Kenya healthcare worker force. I wonder if removing the Kenya portion of the study would make this study stronger and more applicable. | We acknowledge this in the limitations section. However, instead of completely excluding the Kenya sample, we provided stratified results by country and data collection phases as, shown in the supplemental tables. As noted, these results are consistent with the combined sample, except for the higher p-values for Kenya due to the smaller sample size. | Supplemental tables  Pages 31-32 |
| h. The authors also need to explain why they did not find a difference between job satisfaction, gender and type of healthcare facility.  How many facilities in Kenya were recruited and are these private, governmental or other hospitals? Resources at different facilities differ and should be a limitation of the study as well. | We have added a paragraph in the discussion on the lack of significant associations between job satisfaction and gender and the type of healthcare facility.  As noted, providers were recruited using online platforms and not from specific facilities, and so we are unable to report on the number of facilities providers were recruited from. However, the distribution of facilities for each setting is shown in table 1 and highlights the variety and types of different facilities. Table 1 shows that for Kenya, 12% of the providers worked at teaching hospitals, 66% at other government health facilities, and 20% in private faculties. We acknowledge that different resources in the facilities could have influenced the outcomes. But the effect of these resources are likely accounted for by more proximal factors such as preparedness, stress, burnout, etc., which we note in the discussion. | Page 14, 30 |
| i. I think the introductions could be improved better with additional information on what other studies have shown and the lack of data in this area. Also the discussion needs more work and I would request that authors to highlight clearly all their impactful findings and explain them with a justification. | We have expanded on both the introduction and discussion sections to illustrate the impact of these findings. We also included additional references, and provided a revised literature review to respond to these comments. | Page 5, 7, 28-30 |
| j. The authors makes suggestion of how to mitigate their findings in the last sections of the manuscript. This needs clarity and in depth explanation of what they are exactly suggesting to be impactful to the readers especially in low resources areas. What are some of the suggested strategies for the administration to better appreciate staff and improve communication? This blanket statement might be more meaningful if we can adopt ideas implement elsewhere. | We thank the reviewer for this comment. We provided the types of appreciation and effective communication in the revised manuscript, in the implications section. | Page 28-30 |
|  |  |  |
| Journal Requirements:   Please review your reference list to ensure that it is complete and correct. If you have cited papers that have been retracted, please include the rationale for doing so in the manuscript text, or remove these references and replace them with relevant current references. Any changes to the reference list should be mentioned in the rebuttal letter that accompanies your revised manuscript. If you need to cite a retracted article, indicate the article’s retracted status in the References list and also include a citation and full reference for the retraction notice. | We have reviewed the reference list for accuracy and completeness. Additional references were added in response to the reviewer comments | Page 35 |
